# Association between chronic conditions and health-related quality of life: differences by level of urbanization in Peru

**DOI:** 10.1007/s11136-017-1649-7

**Published:** 2017-07-15

**Authors:** Alvaro Taype-Rondan, Elizabeth Sarah Abbs, Maria Lazo-Porras, William Checkley, Robert H. Gilman, Liam Smeeth, J. Jaime Miranda, Antonio Bernabe-Ortiz

**Affiliations:** 1 CRONICAS Center of Excellence in Chronic Diseases, Universidad Peruana Cayetano Heredia, Armendáriz 497, Miraflores, 18 Lima, Peru; 2 School of Medicine and Public Health, University of Wisconsin, Madison, WI USA; 3 Division of Pulmonary and Critical Care, School of Medicine, Johns Hopkins University, Baltimore, MD USA; 4 Department of International Health, Johns Hopkins Bloomberg School of Public Health, Baltimore, MD USA; 5 Faculty of Epidemiology and Population Health, London School of Hygiene and Tropical Medicine, London, UK

**Keywords:** Health-related quality of life, Multiple chronic conditions, Burden of disease, Depressive mood

## Abstract

**Purpose:**

To evaluate the role of urbanization as an effect modifier for the association between specific chronic conditions and number of conditions with health-related quality of life (QOL).

**Methods:**

We analyzed cross-sectional data from the CRONICAS Cohort Study conducted in Lima (highly urbanized), Tumbes (semi-urban), as well as rural and urban sites in Puno. Exposures of interest were chronic bronchitis, depressive mood, hypertension, type 2 diabetes, and a composite variable aggregating the number of chronic conditions (the four exposures plus heart disease and stroke). QOL outcomes were assessed with EuroQol’s EQ-5D visual analogue scale (EQ-VAS). We fitted linear regressions with robust variance to evaluate the associations of interest. Study site was assessed as a potential effect modifier using the likelihood-ratio (LR) test.

**Results:**

We evaluated data on 2433 subjects: 51.3% were female, mean age was 57.2 years. Study site was found to be an effect modifier only for the association between depressive mood and EQ-VAS score (LR test *p* < 0.001). Compared to those without depressive mood, participants with depressive mood scored −13.7 points on the EQ-VAS in Lima, −7.9 in urban Puno, −11.0 in semi-urban Tumbes, and −2.7 in rural Puno. Study site was not found to be an effect modifier for the association between the number of chronic conditions and EQ-VAS (LR test *p* = 0.64).

**Conclusion:**

The impact of depressive mood on EQ-VAS was larger in urban than in rural sites, while site was not an effect modifier for the remaining associations.

**Electronic supplementary material:**

The online version of this article (doi:10.1007/s11136-017-1649-7) contains supplementary material, which is available to authorized users.

## Introduction

Health-related quality of life (QOL) is defined as “an individual’s or a group’s perceived physical and mental health over time” [1]. QOL is worse in patients with chronic non-communicable conditions, who may face a multitude of clinical, psychosocial, and economic challenges [2–4]. Consequently, poorer QOL scores are observed with each additional chronic condition [4–11]. Although not all chronic conditions affect QOL in the same magnitude, the list of conditions notably affecting QOL includes chronic pain disorders and psychiatric conditions [4, 12, 13].

The impact of chronic conditions on QOL could be altered by urbanization level. In rural areas, barriers to healthcare access may challenge clinical follow-up and disease management [14–16], translating to lower QOL scores than those in urban sites with more access to medical care. On the other hand, individuals in rural areas do have higher indices of social support [17] which could promote better QOL and thus balance the effect of lower access to healthcare services in these areas. In addition, differences in culture and customs between areas of different urbanization levels could further modify how chronic conditions impact health-related QOL.

Understanding how chronic conditions affect QOL, particularly across different cultural contexts and regions might help to evaluate the relative impact of chronic conditions, prioritize conditions within limited-resources sites, and design site-specific interventions to improve QOL across different contexts [18]. To date, the majority of studies assessing the association between chronic conditions and QOL have been conducted in urban areas, and we could not find studies exploring how urbanization modifies the chronic condition-QOL association.

Therefore, this study uses data from four sites in Peru with varying degrees of urbanization, with the objective to evaluate the role of urbanization as an effect modifier for the association between specific chronic conditions and number of conditions with health-related QOL.

## Methods

### Study design and sites

This is a secondary analysis using data derived from the CRONICAS Cohort Study, conducted in four diverse sites in Peru: (1) Las Pampas de San Juan de Miraflores, a highly urbanized site in Lima, Peru’s capital, located at sea level with ~15,000 people/km^2^. (2) Urban Puno, a city with approximately 150,000 inhabitants located at 3825 m above sea level in the Peruvian Andes. (3) Semi-urban costal site in Tumbes, located at sea level in north Peru near the Equator, composed of a group of communities with about 20,000 people spread over 80 km^2^. (4) Rural Puno, composed of a group of rural villages surrounding urban Puno [19].

### Participants

At baseline in 2010, the CRONICAS Cohort Study recruited individuals with a minimum age of 35 years who were full-time residents of the selected population sites. A single random selection of subjects was performed at each site, stratified by sex and age, using the most updated census data available. Only one participant per household was enrolled, as detailed elsewhere [19]. The first and second follow-up assessments were completed, on average, 15 and 30 months after baseline. For the present study, we only included participants who completed the second follow-up as the outcome of interest was evaluated in this assessment, and included those who had provided all the variables of interest.

### Procedures

A detailed questionnaire was administrated by trained fieldworkers including socio-demographic characteristics, self-report of chronic conditions, and the Center for Epidemiological Studies Depression Scale [20].

Systolic and diastolic blood pressures were measured three times using an automatic monitor OMRON HEM-780 after 5 min of resting period, and the mean of the last two measurements were used. In addition, fasting blood glucose samples were obtained and processed using an enzymatic colorimetric method (GOD-PAP; Modular P-E/Roche-Cobas, Grenzach-Whylen, Germany) and analyzed at an off-site facility in Lima, following standard laboratory techniques and quality control procedures as detailed elsewhere [19].

### Outcome: quality of life

QOL was assessed with the EuroQol-5D (EQ-5D) questionnaire, which includes the visual analogue scale (EQ-VAS) and a 5-dimensions questionnaire. We used EQ-VAS scores as our main outcome. It assesses perceived QOL by asking participants to rank their current state of wellness from zero, worst imaginable health, to 100, best imaginable health [21, 22].

The 5-dimension questionnaire was our secondary outcome. It evaluates QOL across five health status dimensions: mobility, self-care, usual activities, pain/discomfort, and anxiety/depression. Within each dimension, severity is evaluated with three categories: no problems, some problems, and extreme problems [21, 22]. For our analysis, each dimension was dichotomized as “no problems” and “some or extreme problems.”

We decided not to use the EQ-5D tariffs validated for other Latin American countries because these have not been validated for rural settings where EQ-5D items could have a different impact.

### Exposure: chronic conditions

We included the most prevalent chronic conditions collected in the CRONICAS Cohort Study. Exposures of interest were chronic bronchitis, depressive mood, hypertension (HTN), type 2 diabetes (T2D), and, separately, a composite variable aggregating the number of chronic conditions (the four exposures plus heart disease and stroke). Heart disease and stroke were uncommon in rural Puno, so were not included as single exposures in the analyses.

The rationale for the selection of particular chronic conditions was the availability of objective measurements, validated instruments, as well as conditions with less recall bias. The operational definition of each chronic condition was based in previous references, as detailed in Table 1.

**Table 1:** Definitions of chronic conditions

Chronic condition	Definition
Hypertension	Any of the following: systolic blood pressure (SBP) ≥140 mmHg, or diastolic blood pressure (DBP) ≥90 mmHg, or self-report of physician diagnosis and current use of antihypertensive drugs; according to the seventh report of the Joint National Committee on Prevention, Detection, Evaluation, and Treatment of High Blood Pressure [23]
Chronic bronchitis	Presence of phlegm production on most days for at least 3 months a year, in the last 12 months, similar to previous studies [24]
Depressive mood	A score of ≥23 in the Spanish-validated version of the Center for Epidemiological Studies Depression Scale [20]
Type 2 diabetes	Any of the following conditions: fasting blood glucose ≥126 mg/dL, or self-report of physician diagnosis and current use of diabetes medication; according to the World Health Organization definitions [25]
Heart disease	Self-report physician diagnosis of heart failure or previous myocardial infarction
Stroke	Self-report of previous stroke, diagnosed by a physician

### Effect modifier: study site

Study site, which refers to the site where participants lived, was categorized according to gradient of urbanization as follows: highly urban Lima, urban Puno, semi-urban Tumbes, and rural Puno.

### Other variables

Other variables included in the analyses were sex, age (categorized as <45, 45–54, 55–64, and ≥65 years), educational level (primary or less: 0–6 years, secondary: 7–11 years, and superior: ≥12 years), and wealth index (in tertiles). The wealth index was constructed based on household income, assets, and household facilities as suggested elsewhere, with higher scores indicating greater levels of wealth [26].

### Statistical analyses

For descriptive analysis, means, standard deviations (±SD), absolute and relative frequencies were used. Chi square and ANOVA tests were used to compare the four sites with respect to sex, age, educational level, wealth index, number of chronic conditions, specific chronic conditions, EQ-VAS, and EQ-5D dimensions.

We generated linear regression models with robust variance to calculate coefficients (*β*) and their 95% confidence intervals (95% CI), to evaluate the association between five exposures: HTN, chronic bronchitis, depressive mood, T2D, and number of chronic conditions; and EQ-VAS scores as a numeric outcome. These associations were calculated for the overall population, and for each study site. To answer our research question, study site was assessed as a potential effect modifier using the likelihood-ratio (LR) test.

For our secondary analyses, we generated Poisson regression models with robust variance to calculate prevalence ratios (PR) and 95% CI to evaluate the association between number of chronic conditions and each of the five EQ-5D dimensions for each of the study sites. Both linear and Poisson models were adjusted for sex, age, educational level, and wealth index.

### Ethics

Due to high illiteracy rates, especially in rural areas, all participants provided verbal informed consent. The study was approved by the Institutional Review Boards at Universidad Peruana Cayetano Heredia and A.B. PRISMA, in Lima, Peru, and at the Johns Hopkins Bloomberg School of Public Health in Baltimore, USA.

## Results

### Population characteristics

At baseline, 3601 participants were recruited. Of these, 38 died and 865 were lost during follow-up; thus, only 2698 (74.6%) participants were included in the current study. We further excluded 265 participants with incomplete chronic disease and/or QOL information. Therefore, our current analysis includes data from 2433 participants (67.6% of the original sample enrolled). Participants included in the analysis were more likely to be from Lima and Tumbes, be younger, have a secondary-school education, and be from the highest wealth index, compared to those not included (Supplementary Table 1).

Of the 2433 participants included, 891 (36.6%) were from Lima, 357 (14.7%) from urban Puno, 890 (36.6%) from Tumbes, and 295 (12.1%) from rural Puno. Mean age was 57.2 (SD ± 12.3) years, and 1248 (51.3%) were female (Table 2).

**Table 2:** Descriptive characteristics of the population according to site

Characteristics	Total (*N* = 2433)	Urban Lima (*N* = 891)	Urban Puno (*N* = 357)	Semi-urban Tumbes (*N* = 890)	Rural Puno (*N* = 295)	*P* ^a^
Sex						0.660
Female	1248 (51.3)	471 (52.9)	182 (51.0)	444 (49.9)	151 (51.2)	
Male	1184 (48.7)	420 (47.1)	175 (49.0)	446 (50.1)	143 (48.5)	
Age (years)						0.481
36–44	434 (17.8)	156 (17.5)	59 (16.5)	165 (18.5)	54 (18.3)	
45–54	655 (26.9)	250 (28.1)	103 (28.9)	230 (25.8)	72 (24.4)	
55–64	664 (27.3)	256 (28.7)	94 (26.3)	226 (25.4)	88 (29.8)	
≥65	673 (27.7)	226 (25.4)	101 (28.3)	265 (29.8)	81 (27.5)	
Educational level						<0.001
Primary or less	1053 (43.3)	362 (40.6)	40 (11.2)	479 (53.8)	172 (58.3)	
Secondary	849 (34.9)	371 (41.6)	93 (26.1)	282 (31.7)	103 (34.9)	
Superior	529 (21.7)	157 (17.6)	224 (62.7)	128 (14.4)	20 (6.8)	
Wealth index						<0.001
Lowest tertile	678 (27.9)	111 (12.5)	75 (21.0)	285 (32.0)	207 (70.2)	
Middle tertile	850 (34.9)	329 (36.9)	78 (21.8)	362 (40.7)	81 (27.5)	
Highest tertile	905 (37.2)	451 (50.6)	204 (57.1)	243 (27.3)	7 (2.4)	
Chronic conditions						
Hypertension	573 (23.6)	184 (20.7)	45 (12.6)	317 (35.6)	27 (9.2)	<0.001
Chronic bronchitis	309 (12.7)	87 (9.8)	72 (20.2)	42 (4.7)	108 (36.6)	<0.001
Depressive mood	309 (12.7)	141 (15.8)	45 (12.6)	55 (6.2)	68 (23.1)	<0.001
Type 2 diabetes	254 (10.4)	82 (9.2)	33 (9.2)	124 (13.9)	15 (5.1)	<0.001
Heart disease	131 (5.4)	64 (7.2)	28 (7.8)	37 (4.2)	2 (0.7)	<0.001
Stroke	20 (0.8)	13 (1.5)	2 (0.6)	4 (0.4)	1 (0.3)	0.069
Number of chronic conditions						<0.001
0	1281 (52.7)	499 (56.0)	202 (56.6)	456 (51.2)	124 (42.0)	
1	796 (32.7)	260 (29.2)	95 (26.6)	312 (35.1)	129 (43.7)	
2	276 (11.3)	87 (9.8)	51 (14.3)	103 (11.6)	35 (11.9)	
≥3	80 (3.3)	45 (5.1)	9 (2.5)	19 (2.1)	7 (2.4)	
EQ-visual analogue score^a^	69.4 ± 16.1	71.9 ± 17.3	67.1 ± 16.5	70.7 ± 13.5	60.4 ± 15.6	<0.001
EQ-5D dimensions						
Pain/discomfort	939 (38.6)	389 (43.7)	166 (46.5)	224 (25.2)	160 (54.2)	<0.001
Mobility	427 (17.6)	186 (20.9)	51 (14.3)	113 (12.7)	77 (26.1)	<0.001
Anxiety/depression	378 (15.5)	184 (20.7)	82 (23.0)	45 (5.1)	67 (22.7)	<0.001
Usual activities	198 (8.1)	77 (8.6)	29 (8.1)	41 (4.6)	51 (17.3)	<0.001
Self-care	64 (2.6)	26 (2.9)	7 (2.0)	11 (1.2)	20 (6.8)	<0.001

^a^ *p* values were calculated using *χ* ^2^ or ANOVA tests

In terms of single chronic conditions, 573 participants (23.6%) had HTN, 309 (12.7%) had chronic bronchitis, 309 (12.7%) had depressive mood, 254 (10.4%) had T2D, 131 (5.4%) had heart disease, and 20 (0.8%) had a history of stroke. Chronic bronchitis and depressive mood were more prevalent in rural Puno. When chronic conditions were aggregated, 1152 (47.3%) participants had ≥1 chronic condition. The number of chronic conditions and prevalence of individual chronic conditions varied across sites, except for stroke. Mean EQ-VAS score was highest in Lima, and lowest in rural Puno. The prevalence of having some or extreme problems in most of the EQ-5D dimensions was lower in Tumbes and higher in rural Puno (Table 2).

### Association between chronic conditions and EQ-VAS score

When evaluating the relationship between specific chronic conditions and EQ-VAS score (Table 3), we found that participants with depressive mood and chronic bronchitis had significantly lower EQ-VAS scores than those without these conditions, while EQ-VAS scores in participants with HTN and T2D were not different compared with those without these conditions.

**Table 3:** Association of number of chronic conditions and each specific chronic condition with EQ-VAS score, by study site

Exposures	Total (*N* = 2433)	Lima (*N* = 891)	Urban Puno (*N* = 357)	Semi-urban Tumbes (*N* = 890)	Rural Puno (*N* = 295)	LR test *p* ^a^
	*β* (95% CI)	*β* (95% CI)	*β* (95% CI)	*β* (95% CI)	*β* (95% CI)	
Chronic conditions						
Hypertension	−0.4 (−1.9, 1.1)	−**3.2 (**−**6.1,** −**0.3)**	−2.2 (−7.7, 3.3)	−1.6 (−3.5, 0.4)	2.1 (−3.3, 7.5)	0.66
Chronic bronchitis	−**5.7 (**−**7.6,** −**3.8)**	−1.8 (−5.4, 1.8)	−**4.2 (**−**7.8,** −**0.6)**	−4.3 (−9.2, 0.7)	−3.5 (−7.4, 0.4)	0.59
Depressive mood	−**10.5 (**−**12.8,** −**8.2)**	−**13.7 (**−**17.2,** −**10.2)**	−**7.9 (**−**14.3,** −**1.4)**	−**11.0 (**−**15.2,** −**6.9)**	−2.7 (−8.3, 2.9)	**<0.001**
Type 2 diabetes	−1.3 (−3.6, 0.9)	−2.9 (−7.6, 1.8)	2.1 (−4.5, 8.7)	−2.3 (−4.8, 0.2)	−1.7 (−12.5, 9.2)	0.44
Number of chronic conditions	−3.4 (−4.3, −2.5)	−4.1 (−5.6, −2.6)	−3.3 (−5.4, −1.3)	−2.7 (−4.1, −1.4)	−2.3 (−5.1, 0.4)	0.64

We used linear regression models with robust variances. All models were adjusted for sex, age, education status, and wealth index. Stroke and heart disease were not evaluated due to the small number of cases

Significant associations (*p* < 0.05) are bolded

^a^Likelihood-ratio test to assess the potential effect modifier of study site

Study site was an effect modifier of the association between having depressive mood and EQ-VAS score (LR test *p* < 0.001), but it was not found to be an effect modifier for the association between EQ-VAS score and having HTN, chronic bronchitis, and T2D. Therefore, compared to those without depressive mood, participants with depressive mood scored −13.7 points on the EQ-VAS in Lima, −7.9 in urban Puno, −11.0 in semi-urban Tumbes, and −2.7 in rural Puno.

**Fig. 1:**
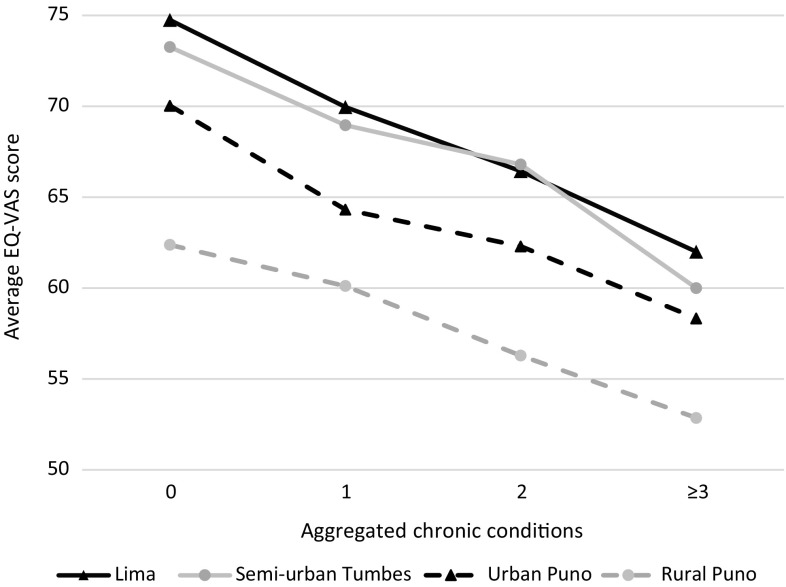
Average EQ-VAS score per number of chronic condition in four study sites

Across all sites, EQ-VAS score decreased with each additional chronic condition (Fig. 1). In the adjusted linear regression, EQ-VAS score was 3.4 points lower for each additional chronic condition. Study site was not found to be an effect modifier in this association (LR test *p* = 0.64) (Table 3).

### Association between number of chronic conditions and EQ-5D dimensions

In multivariable models, we found that the number of chronic conditions was associated with all EQ-5D dimensions in the sample as a whole. The highest PR was for “self-care” (PR = 1.98, 95% CI 1.57–2.49), followed by “anxiety/depression” (PR = 1.67, 95% CI 1.53–1.81). Study site was not an effect modifier for these associations (Table 4).

**Table 4:** Association of number of chronic conditions with EQ-5D dimensions, by study site

Outcomes	Total (*N* = 2433)	Lima (*N* = 891)	Urban Puno (*N* = 357)	Semi-urban Tumbes (*N* = 890)	Rural Puno (*N* = 295)	LR test *p* ^a^
	PR (95% CI)	PR (95% CI)	PR (95% CI)	PR (95% CI)	PR (95% CI)	
Pain/discomfort	1.23 (1.17–1.30)	1.19 (1.11–1.28)	1.14 (1.02–1.27)	1.43 (1.29–1.60)	1.12 (0.99–1.26)	0.08
Mobility	1.38 (1.27–1.50)	1.40 (1.25–1.58)	1.41 (1.14–1.73)	1.25 (1.04–1.50)	1.50 (1.25–1.80)	0.67
Anxiety/depression	1.67 (1.53–1.81)	1.66 (1.48–1.85)	1.42 (1.20–1.69)	2.23 (1.71–2.92)	1.54 (1.26–1.89)	0.16
Usual activities	1.57 (1.38–1.78)	1.57 (1.29–1.93)	1.44 (1.10–1.88)	1.74 (1.33–2.27)	1.44 (1.15–1.81)	0.77
Self-care	1.98 (1.57–2.49)	1.70 (1.16–2.49)	2.81 (1.18–6.71)	3.61 (2.07–6.30)	1.80 (1.04–3.10)	0.36

All models were adjusted for sex, age, education status, and wealth index

^a^Likelihood-ratio test to assess the potential effect modification of study site

## Discussion

### Main findings

Depressive mood and chronic bronchitis, but not HTN nor T2D, were associated with lower EQ-VAS scores. Site was not an effect modifier for the association between EQ-VAS score and having HTN, chronic bronchitis, and T2D. However, site was an effect modifier of the association between EQ-VAS and depressive mood: the EQ-VAS score was lower in people with depressive mood than in those without depressive mood, but this difference was larger in Lima than in rural Puno with a clear pattern of a gradient in the magnitudes of difference in EQ-VAS scores observed across the range of urbanization profiles. Regarding the number of chronic conditions, it was inversely associated with EQ-VAS scores. The association between number of chronic conditions and EQ-VAS scores was similar across all study sites.

### Chronic conditions and EQ-VAS score

Depressive mood was the chronic condition that seemed to have the larger impact in QOL, similar to studies in Finland [8], Canada [11], Spain [27], and the United States [28]. However, since these studies were also cross-sectional, it is possible that, at least in some cases, depressive mood could be the cause rather than the consequence of low QOL. Thus, longitudinal approaches are needed to identify the real impact of depressive mood in QOL.

Chronic bronchitis also had a high impact on QOL, as found in studies that assessed chronic pulmonary conditions (chronic bronchitis, asthma, COPD) [7, 29, 30], which is attributed to the impact of chronic cough, shortness of breath, and phlegm production [7, 31–34]. This suggests that chronic bronchitis should be prioritized in primary care programs in our sites.

EQ-VAS score in persons with T2D was on average 1.3 points lower than in those without T2D. Conversely, in a population study in Korea, EQ-VAS score was 4.6 points lower in persons who self-reported T2D than those who did not [35]. This variation may be due to our inclusion of not only self-reported T2D cases, but also of those with abnormal point-of-care fasting blood glucose. As expected, the very nature of having T2D or hypertension, be it a short duration with the condition or having them without complications, could explain the little impact of these conditions on QOL found in our study [36, 37]. To evaluate this hypothesis, we made a post hoc linear regression in our population, and found that those receiving a prescription for T2D medications (i.e., those with a T2D diagnosis) scored 2.6 points lower on the EQ-VAS than those not on hypoglycemic agents (*p* = 0.10). This suggests that QOL was diminished among participants who were aware of their diagnosis, although other variables such as aware of the gravity and the complications of T2D could also play a role in subjective QOL.

### Number of chronic conditions and EQ-VAS score

For each additional chronic condition, our participants showed a 3.4-unit reduction in their EQ-VAS score. This reduction is substantial, since data from a 5-year cohort population study of elderly Italians found that one unit decrease in EQ-VAS was associated to a 1% increase in mortality and hospitalization rates [38]. As such, strategies to improve QOL in persons with chronic conditions are needed. Increasing healthcare access for physician visits, incorporating disease self-management skills [39], and designing individualized patient regimen with consideration of treatment side effects [40] could potentially decrease treatment burden and therefore improve QOL.

EQ-VAS score was 3.4 points lower for each additional chronic condition. In comparison, a previous study in the United Kingdom found that EQ-VAS score was 5.9 points lower for each additional chronic condition [9]. This study evaluated six chronic conditions: HTN, T2D, cerebrovascular disease, ischemic heart disease, COPD, and asthma, but it did not evaluated depressive mood, which had the highest impact in QOL in our study. Thus, it is possible that the EQ-VAS score per chronic condition would be even lower in this UK study if depressive mood had been included. Then, there could be a great difference between this study and ours in terms of QOL affectation by chronic conditions. This could be explained by the severity of the United Kingdom subjects, who were registered with general practitioners who probably see patients with more advanced complications of their chronic diseases.

### Number of chronic conditions and EQ-VAS score per site

Average EQ-VAS score was higher in Lima and semi-urban Tumbes, intermediate in urban Puno, and lower in rural Puno. This linearly reflects the degree of urbanization, with the exception of semi-urban Tumbes, with values similar to urban Lima. It is possible that semi-urban Tumbes dwellers have strong social or familiar support, similar to those in rural areas as described elsewhere [41], while at the same time having better healthcare access, similar to urban areas. This could also explain the low depressive mood prevalence in this semi-urban site.

Lower QOL values in the rural site could be due to less education, poor economic status, and limited access to health services; all increasing their risk of developing severe health complications [14–16]. In addition, people living in rural areas are more likely to be involved in manual labor and may require more physical skills to perform their usual activities; thus, the decrease of physical capabilities in rural dwellers could have a greater impact on subjective wellbeing than in urban dwellers. Such regional observations would suggest that a higher number of chronic diseases would more dramatically affect QOL in rural than urban sites. However, our results show that EQ-VAS scores per number of chronic conditions were similar across studied sites.

This is probably because the average EQ-VAS score in those without chronic conditions was higher in Lima than in rural Puno (75 and 62 points, respectively). Due to the subjective nature of the EQ-VAS assessment, the same condition could cause a larger decrease in QOL in Lima than in rural Puno [42]. If this is true, it would be incorrect to compare linear regression coefficients with EQ-VAS; therefore, future studies should evaluate this hypothesis comparing EQ-VAS with other more objective QOL questionnaires. Another potential explanation is that rural dwellers may have a higher emotional resilience, which could diminish the subjective effect of chronic conditions on their QOL, as seen in the lower impact of depressive mood on EQ-VAS in rural sites. This should be more thoroughly studied to better understand what factors predict and define QOL for people living in rural versus urban sites.

However, few studies have evaluated the differences of social support, familial support, healthcare access, education, economic status, and labor characteristics, across Peruvian sites, and especially in sites with a heterogeneity of urbanization degree patterns. Moreover, there is a paucity of information about the impact of these variables in objective and subjective QOL. Understanding what levels or profiles of urbanization are directly linked with be deeply studied in order to understand QOL across different sites warrants further evaluation.

### Number of chronic conditions and EQ-5D dimensions

The most prevalent EQ-5D dimension was pain and the least prevalent was self-care, similar to previous studies [9, 11, 43]. All dimensions but anxiety/depression had a higher prevalence in the rural site, while the lowest prevalence across all EQ-5D dimensions was observed in semi-urban Tumbes. This could relate to the rural contextual features and the Tumbes familiar/social support described above.

### Strength and limitations

This study compared the association of multiple and specific chronic conditions and QOL within urban and rural sites. Its results might help us to understand how health-related QOL varies among intra-national sites.

Limitations of our study are as follows: (1) not all chronic conditions with chief impact on QOL were included, such as cancer, chronic pain, and arthritis [10, 11], so that the inclusion of these conditions could increase the impact of number of chronic conditions on EQ-VAS. (2) Some of our diagnostic criteria for chronic conditions are not the current gold standard, such as blood pressure measure in a single day for HTN, and the evaluation of a single fasting blood glucose sample for T2D. In addition, our chronic bronchitis diagnosis was based on symptoms presented during the last 12 months while other studies required symptom persistence for at least 2 years to consider a diagnosis of chronic bronchitis [24]. These evaluations could be over-estimating the prevalence of some chronic conditions, although they could be more accurate than the self-report used in similar previous studies [35–37]. (3) Diminished sample representativeness, especially in older and poor participants, could be underestimating the association, since these subjects could have a greater QOL decrease due to insufficient care of their chronic conditions. (4) Low sample size, especially in rural Puno, which could affect the extrapolation of our results to this site.

## Conclusion

Depressive mood, chronic bronchitis, and the number of chronic conditions were related to lower EQ-VAS scores. The associations between HTN, chronic bronchitis, T2D, and number of chronic conditions with EQ-VAS score were similar across sites with different levels of urbanization, while depressive mood had a larger impact on EQ-VAS score for participants living in urban sites than those living in rural sites. This suggests that urbanization could influence the impact that some chronic conditions have on health-related QOL, and warrants attention to context when evaluating and comparing QOL across different sites.

## Electronic supplementary material

Below is the link to the electronic supplementary material.

Supplementary material 1 (DOCX 19 kb)
