# Supplementary material for: Association between chronic conditions and health-related quality of life: differences by level of urbanization in Peru
Source: Qual Life Res. 2017 Jul 15;26(12):3439–47. doi: 10.1007/s11136-017-1649-7 (PMC5681970; doi:10.1007/s11136-017-1649-7)
Supplement: Supplementary file 1 — Supplementary material 1 (DOCX 19 kb) [file 11136_2017_1649_MOESM1_ESM.docx]

## Supplementary table 1. Comparison between participants included and not included in the analysis, from the total participants colected in the CRONICAS baseline study

| **Characteristics** | **Not included N=1168** | **Included N=2433** | **p** |
| --- | --- | --- | --- |
| Site |  |  | <0.001 |
| Lima | 214 (19.4) | 891 (80.6) |  |
| Urban Puno | 407 (53.3) | 357 (46.7) |  |
| Semi-urban Tumbes | 142 (13.8) | 890 (86.2) |  |
| Rural Puno | 405 (57.9) | 295 (42.1) |  |
| Sex |  |  | 0.766 |
| Female | 604 (32.6) | 1248 (67.4) |  |
| Male | 561 (32.1) | 1184 (67.9) |  |
| Age (years) |  |  | 0.003 |
| 36 to 44 | 258 (30.1) | 600 (69.9) |  |
| 45 to 54 | 294 (31.9) | 628 (68.1) |  |
| 55 to 64 | 263 (28.7) | 654 (71.3) |  |
| ≥65 | 349 (38.8) | 551 (61.2) |  |
| Educational level |  |  | <0.001 |
| Primary or less | 590 (35.9) | 1053 (64.1) |  |
| Secondary | 306 (26.5) | 849 (73.5) |  |
| Superior | 271 (33.9) | 529 (66.1) |  |
| Wealth index |  |  | <0.001 |
| Lowest tertile | 523 (43.5) | 678 (56.5) |  |
| Middle tertile | 351 (29.2) | 850 (70.8) |  |
| Highest tertile | 294 (24.5) | 905 (75.5) |  |
| **Chronic conditions in the baseline** |  |  |  |
| Hypertension |  |  | 0.375 |
| No | 645 (24.8) | 1958 (75.2) |  |
| Yes | 171 (26.5) | 475 (73.5) |  |
| Chronic bronchitis |  |  | 0.172 |
| No | 693 (23.5) | 2253 (76.5) |  |
| Yes | 54 (27.8) | 140 (72.2) |  |
| Type 2 diabetes |  |  | 0.189 |
| No | 1113 (32.6) | 2297 (67.4) |  |
| Yes | 53 (28.0) | 136 (72.0) |  |
| Heart disease |  |  | 0.000 |
| No | 1142 (32.9) | 2325 (67.1) |  |
| Yes | 24 (18.2) | 108 (81.8) |  |
| Stroke |  |  | 0.662 |
| No | 1160 (32.4) | 2423 (67.6) |  |
| Yes | 6 (37.5) | 10 (62.5) |  |

Depressive mood could not be evaluated because it was not assessed in the baseline.
